# Triglyceride-glucose index mediates the association between residual cholesterol and stroke among middle-aged and older adults in China: a prospective, nationwide, population-based study

**DOI:** 10.3389/fcvm.2024.1429993

**Published:** 2024-12-05

**Authors:** Xu Li, Jia-Guang Hu, Qian Liao, Ying Wu, Rong-Rui Huo

**Affiliations:** ^1^ Guangxi Health Commission Key Laboratory of Clinical Biotechnology, Liuzhou People’s Hospital, Liuzhou, China; ^2^ Division of Infectious Diseases, Liuzhou People’s Hospital, Liuzhou, China; ^3^ Department of Epidemiology and Health Statistics, School of Public Health, Guangxi Medical University, Nanning, China; ^4^ Department of Experimental Research, Guangxi Medical University Cancer Hospital, Nanning, China

**Keywords:** stroke, triglyceride-glucose index, residual cholesterol, insulin resistance, CHARLS

## Abstract

**Background:**

Both triglyceride-glucose (TyG) index and residual cholesterol (RC) are predictors of stroke; however, to what extent the RC is associated with stroke through TyG index is unclear. This study examined whether the TyG index mediates the association of RC with incident stroke and the extent of interaction or joint relations of RC and TyG index with stroke in middle-aged and older Chinese adults.

**Methods:**

This is an ongoing prospective cohort study initiated in 2011 that included 10,569 middle-aged and older Chinese adults without stroke at baseline. The exposure was RC, the mediator was TyG index, and the outcome was stroke which followed up from June, 2011, to June, 2018. Mediation analysis was used to explore whether the TyG index mediated the association between RC and stroke risk.

**Results:**

Of the 10,569 participants, 4,978 (47.1%) were men; the mean (SD) age was 59.01 (9.43) years. During a median follow-up of 7.1 years, 734 (7.0%) participants experienced a stroke. In the adjusted Cox models, A one SD increase in RC was associated with an elevated risk of stroke (HR, 1.09; 95% CI, 1.02–1.16), as well as TyG index (HR, 1.14; 95% CI, 1.06–1.23). No significant multiplicative or additive interactions were found between the TyG index and RC on stroke risk (HR for multiplicative: 1.07, 95% CI, 0.67–1.70; Synergy index: 1.05, 95% CI, 0.16–6.88). TyG index mediated the association between RC and stroke (b, −0.16; 95% CI, −0.30 to −0.03). Subgroup analyses and sensitivity analyses showed consistent results.

**Conclusions:**

This study evidence that the TyG index completely mediates the association between RC and stroke risk among middle-aged and older Chinese adults. These findings highlight the importance of considering RC and the TyG index in stroke risk assessment.

## 1 Introduction

Studies have illuminated the intricate connection between residual cholesterol (RC) levels and the risk of stroke (1–4). The term remnant cholesterol describes the cholesterol in triglyceride-rich lipoproteins, composed of very low-density lipoproteins and intermediate-density lipoproteins in the fasting state, and these 2 lipoproteins together with chylomicron remnants in the nonfasting state (5). A recent study has consistently shown that elevated levels of RC are associated with an increased risk of stroke, regardless of low-density lipoprotein (LDL) cholesterol levels (6). These findings have significant implications for stroke prevention and management, which has caused an enormous burden given the high morbidity and mortality rates (7, 8). The underlying mechanisms linking RC to stroke are multifaceted, encompassing direct and indirect pathways. Elevated RC levels, particularly when accompanied by atherogenic remnants, contribute to atherosclerosis, endothelial dysfunction, and systemic inflammation, all of which play critical roles in stroke pathogenesis (9, 10).

Studies have consistently revealed a significant positive association between elevated levels of RC and an elevated triglyceride-glucose (TyG) index, moreover, elevated levels of RC may contribute to the development of the disease by triggering insulin resistance (11). This association highlights the potential role of RC in identifying individuals at heightened risk for stroke, particularly among those with underlying insulin resistance. The TyG index, a novel marker derived from fasting triglycerides and glucose levels, has recently emerged as a significant indicator of insulin resistance for assessing the risk of developing cardiovascular diseases, particularly in settings where more sophisticated measures of insulin resistance are unavailable (12–15). Multiple studies have examined the associations of RC and TyG index with stroke (16–19). However, important gaps remain. First, limited research has been performed on the interaction and joint associations of RC and TyG index with stroke. Second, the TyG index mediates the association between RC and stroke, or vice versa, which is unclear. Third, it remains unclear whether the findings are consistent among subpopulations of different age, sex, and other groups.

To address these gaps, we employed data from the China Health and Retirement Longitudinal Study (CHARLS) to comprehensively evaluate the intricate relationships between RC and the TyG index with stroke. By delving into this complex interplay, we aim to contribute to a more profound comprehension of stroke pathophysiology and to pave the way for novel avenues in the prevention and management of this debilitating condition.

## 2 Methods

### 2.1 Study design and population

This study undertakes a secondary analysis of data derived from the China Health and Retirement Longitudinal Study (CHARLS), a comprehensive nationwide cohort study designed to accurately represent the national population (20). The detailed design and methodology of CHARLS have been expounded upon in prior publications (20). In brief, the study employed a multistage stratified probability-proportional-to-size sampling method to sample 17,708 participants from 10,257 households. Participants were recruited from 150 counties or districts and 450 villages spanning 28 provinces in China between June 2011 and March 2012. Sociodemographic, lifestyle factors, and health-related data were collected using a standardized questionnaire. The initial survey, Wave 1, achieved an 80.5% response rate, with subsequent follow-up assessments conducted biennially: Wave 2 in 2013, Wave 3 in 2015, and Wave 4 in 2018. For the present analysis, only participants aged 45 or above with comprehensive data on TyG and RC were included, excluding those with a previous history of stroke.

The CHARLS study received approval from Peking University's institutional review board, and all participants provided written informed consent. This study adheres to the Strengthening the Reporting of Observational Studies in Epidemiology (STROBE) guidelines (21).

### 2.2 Assessment of TyG index and RC

The Chinese Center for Disease Control and Prevention (CDC), situated in Beijing, expeditiously received venous blood samples within a two-week timeframe from the CDC station. These samples were promptly preserved at −20°C. Following the completion of assays at the laboratory of the Chinese Medical University, they were transferred to a deep freezer maintaining a temperature of −80°C. The Youanmen Clinical Laboratory of Capital Medical University analyzed TG, FPG, total cholesterol (TC), high-density lipoprotein cholesterol (HDL-C), and low-density lipoprotein cholesterol (LDL-C) concentrations using the enzyme colorimetric assay. The TyG index was calculated using the formula ln[TG (mg/dl) × FPG (mg/dl)/2] (22), while the RC was determined by the formula TC (mg/dl)—LDL-C (mg/dl)—HDL-C (mg/dl) (23).

### 2.3 Outcome

The outcome of this study was the incidence of stroke. Consistent with prior research (20, 24), stroke incidents were discerned via self-reports, wherein participants verified a physician's diagnosis of stroke. The recorded date of the stroke diagnosis was delineated as the period between the latest interview date and the date when the stroke incident was reported (20, 24).

### 2.4 Covariates

In the baseline assessment (Wave 1), trained interviewers collected sociodemographic and health-related data using a structured questionnaire. The questionnaire covered age, gender, residence, marital status, and educational level, which was categorized as no formal education, primary school, middle or high school, or college and above. Marital status included two classifications: married or another marital status, encompassing individuals who were never married, separated, divorced, or widowed. Health-related variables encompassed self-reported smoking and drinking status, categorized as never, former, or current, self-reported physician-diagnosed medical conditions (diabetes, hypertension, heart disease, kidney disease, and dyslipidemia), and the use of medications for diabetes, hypertension, and dyslipidemia. Laboratory tests included measurements of estimated glomerular filtration rate (eGFR), high-sensitivity C-reactive protein (hsCRP), and glycosylated hemoglobin, type A1c (HbA1c) (25). Diabetes was defined as fasting plasma glucose ≥126 mg/dl, current use of antidiabetic medication, or self-reported history of diabetes. Dyslipidemia was defined as total cholesterol levels ≥240 mg/dl, triglyceride levels ≥150 mg/dl, low-density lipoprotein cholesterol levels ≥160 mg/dl, high-density lipoprotein cholesterol levels <40 mg/dl, current use of lipid-lowering medication, or self-reported history of dyslipidemia. Hypertension was defined as systolic blood pressure ≥140 mmHg, diastolic blood pressure ≥90 mmHg, ongoing antihypertensive medication use, or a self-reported history of the condition. A trained nurse measured height, weight, and blood pressure, and body mass index was calculated as weight in kilograms divided by height in meters squared. The eGFR was calculated using the 2009 creatinine equation from the Chronic Kidney Disease Epidemiology Collaboration (26).

### 2.5 Statistical analysis

Descriptive statistics were employed to characterize the data, presenting means and standard deviations (SDs) for normally distributed continuous variables and medians with interquartile ranges (IQRs) for non-normally distributed continuous variables. Categorical variables were described using frequencies and percentages. Approximately 19 percent (2,054 out of 10,569) of total data items were identified as missing, presumed to be missing at random, and consequently imputed using the multiple imputations of the chained equations method based on the baseline characteristics.

To examine the associations of TyG index and RC with incident stroke events, both TyG index and RC were split into quartiles and then were included in Cox proportional hazards models with quartile 1 as the reference group. Hazard ratios (HRs) with 95% confidence intervals (CIs) were calculated. Three models were estimated: in model 1, age and gender were adjusted; in model 2, age, gender, marital status, residence, education level, body mass index, smoking status, and drinking status were adjusted; and in model 3, the variables in model 2 plus diabetes, hypertension, heart disease, dyslipidemia, kidney disease, history of medication use for diabetes, history of medication use for hypertension, history of medication use for dyslipidemia, systole blood pressure, diastolic blood pressure, HbA1c, hsCRP, and eGFR were adjusted. In addition, we explored the potential nonlinear associations using 3-knotted restricted cubic spline (RCS) regression. Subgroup analyses were conducted to examine whether the potential associations of TyG index and RC with stroke events were moderated by the demographic and clinical characteristics. *P* values for interaction were evaluated using interaction terms and likelihood ratio tests. Finally, we examined the correlation between the TyG index and RC using the Spearman test.

To assess both additive and multiplicative interactions, we introduced a product term of TyG index (in quartiles) and RC (in quartiles) into the Cox model. The HR with its corresponding 95% CIs for the product term served as the measure of interaction on the multiplicative scale. A statistically significant multiplicative interaction was determined if the CIs did not include 1. For the evaluation of additive interactive effects, we employed the synergy index (SI). This metric provides insights into various aspects of interaction, including the portion of the effect attributable to interaction, the proportion of the combined effect arising from interaction, and the ratio between the combined effect and individual effects. An SI value of 1 indicates an absence of interactive effects between RC and TyG index regarding stroke incidence. Conversely, an SI greater than 1 suggests that the combined effects of RC and TyG index on stroke incidence surpass the sum of their individual effects, indicating synergistic effects. Conversely, an SI less than 1 indicates that the combined effects are smaller than the sum of the individual effects of RC and TyG index. We computed 95% CIs for SI using the delta method, and a statistically significant additive interaction was established if its CIs did not include 1.

We conducted an examination of both indirect associations of RC with stroke, mediated by the TyG index, and direct associations unmediated by the TyG index. Employing the “mediation” package in R, we utilized a regression-based approach to calculate the total effect, natural indirect effects (NIE), and natural direct effects (NDE) of RC on stroke incidence (Supplementary Figure S1). Two models were formulated: one entailed a multivariable linear regression model for the TyG index (as the mediator), conditioned on RC (as the exposure) and confounding variables; the other comprised a multivariable Cox proportional hazard regression model for stroke (as the outcome), conditioned on TyG index, RC, and confounding variables. The NDE conveyed the impact of RC on stroke independently of the TyG index, while the NIE represented the proportion of RC influenced by its connection with the TyG index over time. Subgroup analyses were conducted to test the robustness and potential variations by demographic and clinical characteristics.

Three sensitivity analyses were conducted as follows: (1) repeating all analyses using the complete data set (8,515 participants) without multiple imputations; (2) using the Fine and Gray competing risk model (27) to account for competing risks due to mortality when estimated the associations of TyG index and RC with stroke; and (3) using TyG index at Wave 3 when analyzed the mediating effects to minimize the possibility of reverse causality on the observed associations. Two-sided *P* < 0.05 was considered as statistically significant. All analyses were performed using R statistical software version 4.3.0 (R Foundation).

## 3 Results

### 3.1 Population characteristics

**Figure 1:**
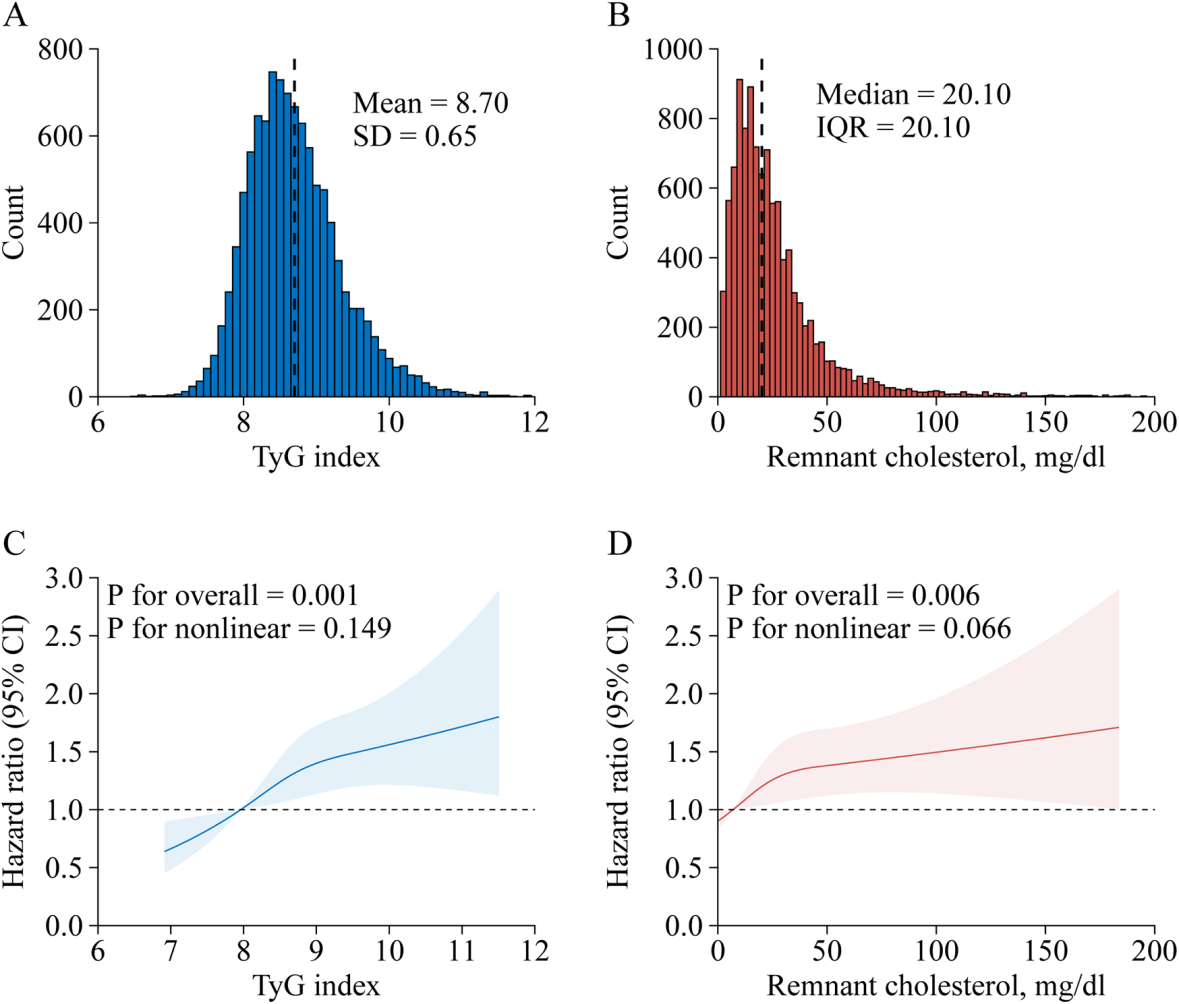
Nonlinear associations of triglyceride-glucose index and remnant cholesterol with stroke risk. Distribution for TyG index **(A)** and remnant cholesterol **(B)**; Graphs show HRs for stroke risk according to TyG index **(C)** and remnant cholesterol **(D)**, all models were adjusted for age, gender, marital status, residence, education level, body mass index, smoking status, and drinking status, diabetes, hypertension, heart disease, dyslipidemia, kidney disease, history of medication use for diabetes, history of medication use for hypertension, history of medication use for dyslipidemia, systole blood pressure, diastolic blood pressure, HbA1c, hsCRP, and eGFR. Data were fitted by a restricted cubic spline Cox proportional hazards regression model. Solid lines indicate HRs, and shadow shapes indicate 95% CIs. CI, confidence interval; IQR, interquartile range; SD, standard deviation; TyG, triglyceride-glucose.

Of the 17,708 CHARLS participants at the study baseline, we excluded 777 individuals younger than 45 years, 634 with stroke at baseline, 5,471 missing TyG index, 21 missing RC, and 236 with extreme RC value. Finally, 10,569 participants were included for analysis. A detailed description of the selection process for the study analytic sample is included in Supplementary Figure S2. A comparison of baseline characteristics between participants included and those who were not included in the analysis was shown in Supplementary Table S1. Of the 10,569 participants, the mean (SD) age at baseline was 59.01 (9.43) years; 4,978 (47.1%) of the participants were men. Table 1 shows the characteristics of the participants. At baseline, the mean (SD) TyG index was 8.70 (0.65) (Figure 1A), while the median (IQR) RC was 20.10 (20.10) mg/dl (Figure 1B). During a median follow-up of 7.1 years, 734 (7.0%) participants experienced an incident stroke.

**Table 1:** Baseline characteristics of 10,596 participants.

Characteristic	Overall
Age, years	
Mean ± SD	59.01 ± 9.43
<60	6,002 (56.8%)
≥60	4,567 (43.2%)
Gender	
Male	4,978 (47.1%)
Female	5,591 (52.9%)
Marital status	
Married	8,736 (82.7%)
Other	1,833 (17.3%)
Residence	
Urban	3,861 (36.5%)
Rural	6,708 (63.5%)
Education level	
No formal education	3,076 (29.1%)
Primary school	4,235 (40.1%)
Middle or high school	2,874 (27.2%)
College or above	384 (3.6%)
Body mass index, kg/m^2^^a^	
<18.5	610 (5.8%)
18.5–23.9	4,677 (44.3%)
24.0–27.9	2,628 (24.9%)
≥28.0	1,036 (9.8%)
Smoking status^a^	
Never	6,387 (60.4%)
Former	883 (8.4%)
Current	3,089 (29.2%)
Drinking status^a^	
Never	6,187 (58.5%)
Former	869 (8.2%)
Current	3,502 (33.1%)
History of comorbidities	
Diabetes^a^	641 (6.1%)
Hypertension^a^	4,193 (39.7%)
Heart disease^a^	1,280 (12.1%)
Dyslipidemia^a^	1,034 (9.8%)
Kidney disease^a^	616 (5.8%)
History of medication use	
Diabetes medications^a^	415 (3.9%)
Hypertension medications^a^	2,080 (19.7%)
Dyslipidaemia medications^a^	518 (4.9%)
Blood pressure, mmHg	
Systolic^a^	129.61 ± 21.46
Diastolic^a^	75.36 ± 12.22
HbA1c, %^a^	5.27 ± 0.82
Median hsCRP (IQR), mg/L	1.04 (0.56, 2.21)
Median eGFR (IQR), ml/min/1.73 m^2^^a^	73.41 (53.75, 96.81)
FBG, mg/dl	110.32 ± 36.92
TC, mg/dl	193.26 ± 37.89
Median triglycerides (IQR), mg/dl	107.08 (76.11, 156.65)
HDL-C, mg/dl	50.84 ± 15.01
LDL-C, mg/dl	116.52 ± 34.77
TyG index^b^	8.70 ± 0.65
RC, mg/dl^c^	20.10 (11.98, 32.09)

Data were presented as mean ± SD or *n* (%), unless otherwise specified.

eGFR, estimated glomerular filtration ratio; FBG, fast blood glucose; HbA1c, glycated hemoglobin; HDL-C, high-density lipoprotein cholesterol; hsCRP, high-sensitivity C-reactive protein; IQR, interquartile range; LDL-C, low-density lipoprotein cholesterol; RC, remnant cholesterol; SD, standard deviation; TC, total cholesterol; TyG, triglyceride-glucose.

^a^ Missing data: 1,618 for body mass index, 210 for smoking status, 11 for drinking status, 96 for diabetes, 1,123 for hypertension, 41 for heart disease, 206 for dyslipidemia, 52 for kidney disease, 97 for history of medication use for diabetes, 59 for history of medication use for hypertension, 213 for history of medication use for dyslipidemia, 1,590 for systole blood pressure, 1,591 for diastolic blood pressure, 92 for HbA1c, 1 for eGFR.

^b^ TyG index was calculated by the formula ln[Triglyceride (mg/dl) × Fasting blood glucose (mg/dl)/2].

^c^ RC was calculated by the formula TC (mg/dl)—LDL-C (mg/dl)—HDL-C (mg/dl).

### 3.2 Association of TyG index with stroke risk

Stroke risk in associations with TyG index categorized into quartiles among participants are presented in Table 2. After adjusting for potential confounders (in model 3), when compared with Quartile 1, the adjusted HRs (95% CIs) for incident stroke were 1.26 (1.00–1.60) for Quartile 2, 1.43 (1.14–1.80) for Quartile 3, and 1.46 (1.16–1.85) for Quartile 4. A one SD increase in TyG index was associated with an elevated risk of stroke (HR, 1.14; 95% CI, 1.06–1.23), and there were no interactions between TyG index and covariates (Table 3). A linear and positive association of TyG index with the risk of incident stroke using RCS regression was also found (for association, *P* = 0.001; for nonlinearity, *P* = 0.169; Figure 1C). Similar results were found when using the Fine and Gray model with death as a competing risk event (Supplementary Table S2), or complete data analyses were conducted (Supplementary Table S3).

**Table 2:** Associations of triglyceride-glucose index and remnant cholesterol with stroke risk.

Variables	No. of event/total	Model 1^a^		Model 2^b^		Model 3^c^	
		HR (95% CI)	*P* value	HR (95% CI)	*P* value	HR (95% CI)	*P* value
TyG index							
Quartile							
Q1 [5.18, 8.25]	122/2,643	Reference		Reference		Reference	
Q2 (8.25, 8.62]	163/2,642	1.36 (1.07–1.72)	0.011	1.31 (1.03–1.65)	0.026	1.26 (1.00–1.60)	0.051
Q3 (8.62, 9.07]	210/2,642	1.76 (1.41–2.21)	<0.001	1.58 (1.26–1.98)	<0.001	1.43 (1.14–1.80)	0.002
Q4 (9.07, 12.3]	239/2,642	2.06 (1.65–2.56)	<0.001	1.76 (1.40–2.20)	<0.001	1.46 (1.16–1.85)	0.002
Per-1-SD increase	734/10,569	1.29 (1.21–1.38)	<0.001	1.22 (1.14–1.31)	<0.001	1.14 (1.06–1.23)	0.001
Remnant cholesterol							
Quartile							
Q1 [0.39, 12.00]	139/2,647	Reference		Reference		Reference	
Q2 (12.00, 20.10]	180/2,643	1.35 (1.08–1.68)	0.008	1.29 (1.03–1.61)	0.026	1.25 (1.00–1.56)	0.052
Q3 (20.10, 32.10]	174/2,642	1.31 (1.05–1.64)	0.018	1.20 (0.96–1.51)	0.104	1.14 (0.91–1.43)	0.252
Q4 (32.10, 195.00]	241/2,637	1.88 (1.52–2.31)	<0.001	1.61 (1.30–1.99)	<0.001	1.45 (1.17–1.80)	0.001
Per-1-SD increase	734/10,569	1.19 (1.13–1.26)	<0.001	1.14 (1.07–1.21)	<0.001	1.09 (1.02–1.16)	0.006

CI, confidence interval; HR, hazard ratio; SD, standard deviation; TyG, triglyceride-glucose; Q, quartile.

^a^ Adjusted for age and gender.

^b^ Adjusted for age, gender, marital status, residence, education level, body mass index, smoking status, and drinking status.

^c^ Adjusted as model 2 plus diabetes, hypertension, heart disease, dyslipidemia, kidney disease, history of medication use for diabetes, history of medication use for hypertension, history of medication use for dyslipidemia, systole blood pressure, diastolic blood pressure, HbA1c, hsCRP, and eGFR.

**Table 3:** Associations of remnant cholesterol and triglyceride-glucose index with stroke risk stratified by different factors^a^.

Subgroup	RC with stroke risk		TyG index with stroke risk	
	HR (95% CI)	P for interaction	HR (95% CI)	P for interaction
Age, years		0.423		0.589
<60	1.09 (1.00–1.19)		1.12 (1.00–1.25)	
≥60	1.07 (0.98–1.18)		1.15 (1.03–1.28)	
Gender		0.265		0.255
Male	1.11 (1.02–1.21)		1.17 (1.05–1.30)	
Female	1.06 (0.97–1.16)		1.10 (0.98–1.23)	
Marital status		0.528		0.368
Married	1.09 (1.02–1.17)		1.14 (1.05–1.24)	
Other	1.08 (0.91–1.28)		1.13 (0.93–1.38)	
Residence		0.332		0.573
Urban	1.13 (1.03–1.24)		1.17 (1.03–1.33)	
Rural	1.06 (0.98–1.16)		1.13 (1.02–1.24)	
Education level		0.615		0.553
No formal education	1.07 (0.95–1.20)		1.18 (1.01–1.36)	
Primary school	1.05 (0.94–1.17)		1.09 (0.97–1.23)	
Middle or high school	1.12 (1.01–1.25)		1.18 (1.01–1.36)	
College or above	1.09 (0.79–1.50)		0.95 (0.61–1.49)	
Body mass index, kg/m^2^		0.578		0.505
<18.5	0.67 (0.31–1.43)		1.13 (0.66–1.94)	
18.5–23.9	1.09 (0.98–1.22)		1.19 (1.06–1.33)	
24.0–27.9	1.04 (0.93–1.15)		1.03 (0.90–1.18)	
≥28.0	1.12 (1.00–1.25)		1.17 (0.99–1.38)	
Smoking status		0.959		0.943
Never	1.10 (1.02–1.20)		1.17 (1.05–1.29)	
Former	1.07 (0.89–1.29)		1.17 (0.93–1.47)	
Current	1.07 (0.95–1.21)		1.08 (0.94–1.24)	
Drinking status		0.069		0.079
Never	1.13 (1.04–1.22)		1.18 (1.06–1.31)	
Former	0.87 (0.66–1.13)		0.92 (0.70–1.21)	
Current	1.08 (0.97–1.20)		1.16 (1.02–1.32)	
Diabetes		0.064		0.208
No	1.06 (0.99–1.14)		1.12 (1.03–1.22)	
Yes	1.26 (1.09–1.46)		1.34 (1.06–1.68)	
Hypertension		0.927		0.897
No	1.09 (0.96–1.24)		1.14 (1.00–1.31)	
Yes	1.09 (1.01–1.17)		1.14 (1.04–1.25)	
Heart disease		0.508		0.140
No	1.10 (1.02–1.17)		1.17 (1.08–1.28)	
Yes	1.06 (0.92–1.22)		1.02 (0.86–1.21)	
Dyslipidemia		0.706		0.745
No	1.08 (1.00–1.17)		1.17 (1.07–1.27)	
Yes	1.10 (0.98–1.23)		1.05 (0.89–1.25)	
Kidney disease		0.643		0.697
No	1.09 (1.02–1.16)		1.15 (1.06–1.24)	
Yes	1.13 (0.88–1.44)		1.07 (0.77–1.47)	

^a^ All models were adjusted for age, gender, marital status, residence, education level, body mass index, smoking status, drinking status, diabetes, hypertension, heart disease, dyslipidemia, kidney disease, history of medication use for diabetes, history of medication use for hypertension, history of medication use for dyslipidemia, systole blood pressure, diastolic blood pressure, HbA1c, hsCRP, and eGFR.

### 3.3 Association of RC with stroke risk

Table 2 shows the associations between TyG index and incident stroke. After adjusting for confounders (in model 3), when compared with Quartile 1, the adjusted HRs (95% CIs) for incident stroke were 1.25 (1.00–1.56) for Quartile 2, 1.14 (0.91–1.43) for Quartile 3, and 1.45 (1.17–1.80) for Quartile 4. A one SD increase in RC was associated with an elevated risk of stroke (HR, 1.09; 95% CI, 1.02–1.16), and there were no interactions between RC and covariates (Table 3). A linear and positive association of RC with the risk of incident stroke using RCS regression was also found (for association, *P* = 0.006; for nonlinearity, *P* = 0.066; Figure 1D). The results did not significantly change when using the Fine and Gray model with death as a competing risk event (Supplementary Table S2), or complete data analyses were conducted (Supplementary Table S3).

### 3.4 Association between RC and TyG index

**Figure 2:**
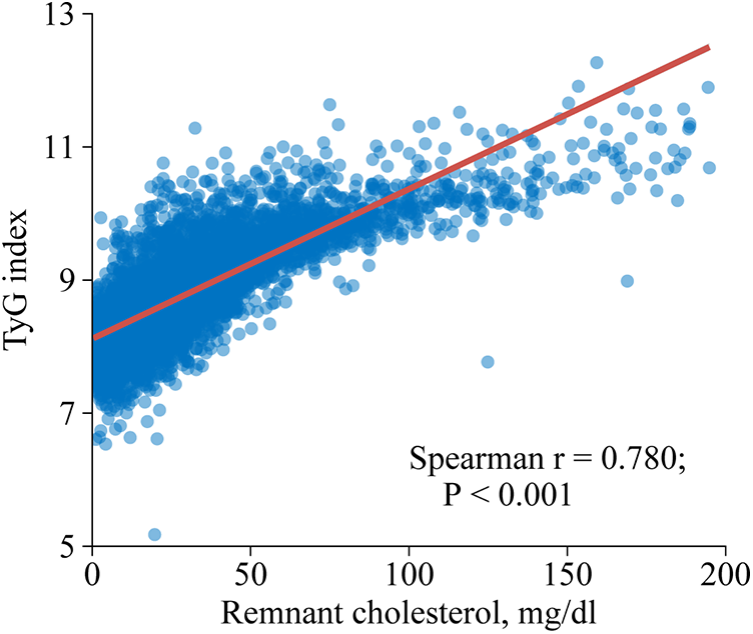
Spearman correlation analysis of remnant cholesterol and triglyceride-glucose index. TyG, triglyceride-glucose.

This study observed a positive association between RC and TyG index as shown in Figure 2 (Spearman *r* = 0.780; *P* < 0.001). Similar results were found when complete data analyses were conducted (Spearman *r* = 0.779; *P* < 0.001; Supplementary Figure S3).

### 3.5 Interaction and joint analysis

**Figure 3:**
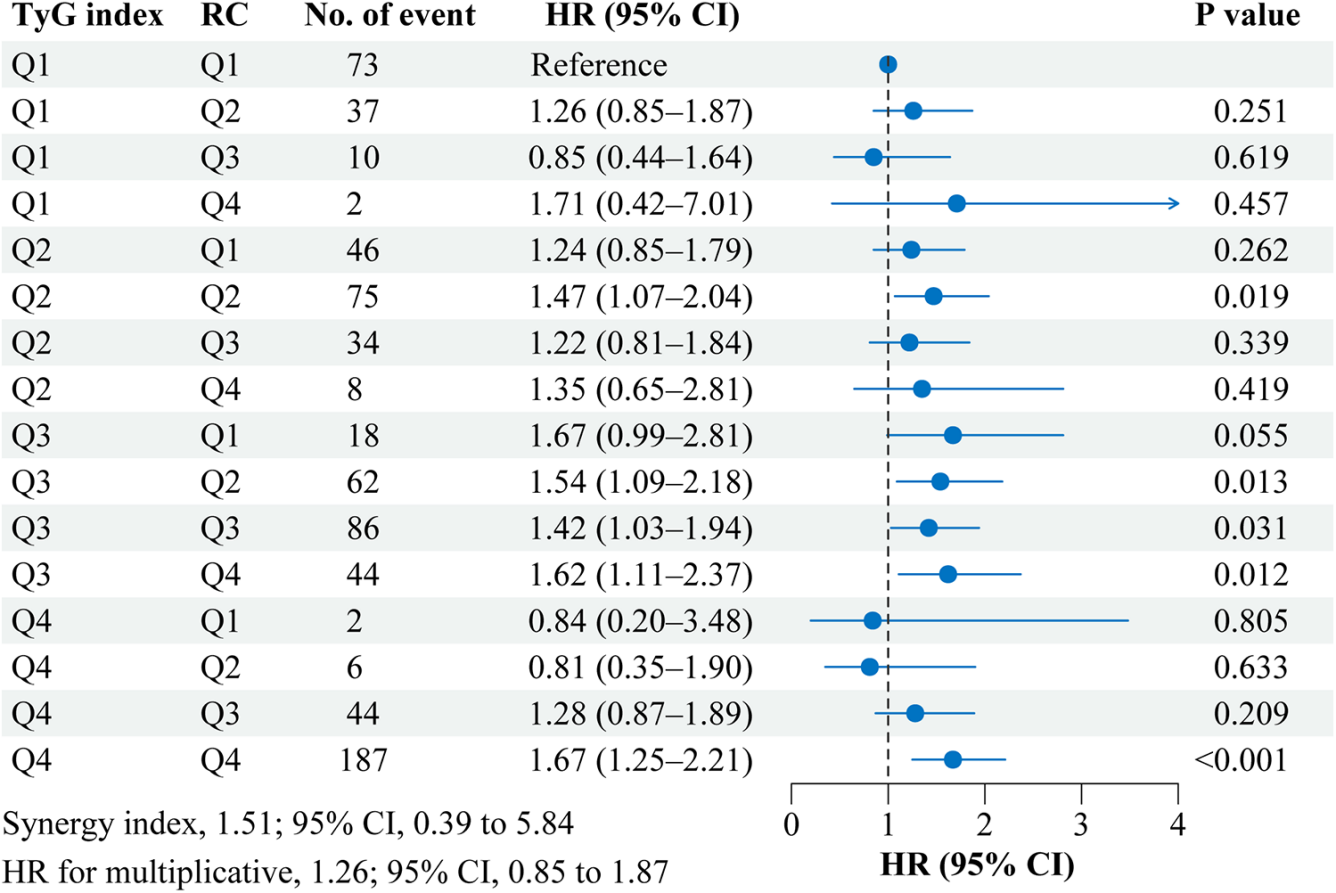
Interaction and joint effects for triglyceride-glucose index and remnant cholesterol on stroke risk. Graphs show the interaction and joint effects of the TyG index and remnant cholesterol with stroke risk. The model was adjusted for age, gender, marital status, residence, education level, body mass index, smoking status, drinking status, diabetes, hypertension, heart disease, dyslipidemia, kidney disease, history of medication use for diabetes, history of medication use for hypertension, history of medication use for dyslipidemia, systole blood pressure, diastolic blood pressure, HbA1c, hsCRP, and eGFR. Additive interaction was evaluated using synergy index (SI) between the TyG index and remnant cholesterol, and the additive interaction was statistically significant when its CI did not include 1. Multiplicative interaction was evaluated using HR for the product term between the TyG index and remnant cholesterol, and the multiplicative interaction was statistically significant when its CI did not include 1. CI, confidence interval; HR, hazard ratio; Q, quartile, RC, remnant cholesterol; TyG, triglyceride-glucose.

Figure 3 shows the interaction and joint association of TyG index and RC on the stroke risk. Compared with participants with the lowest quartile of TyG index (Quartile 1) and RC (Quartile 1), participants with the highest quartile of TyG index (Quartile 4) and RC (Quartile 4) had a 67.0% increased risk of incident stroke (HR, 1.67; 95% CI, 1.25–2.21). However, No significant multiplicative or additive interactions were found between TyG index and RC on stroke risk (HR for multiplicative: 1.07, 95% CI, 0.67–1.70; SI: 1.05, 95% CI, 0.16–6.88). Similar results were found when complete data analyses were conducted (Supplementary Figure S4).

### 3.6 Mediation analyses

**Figure 4:**
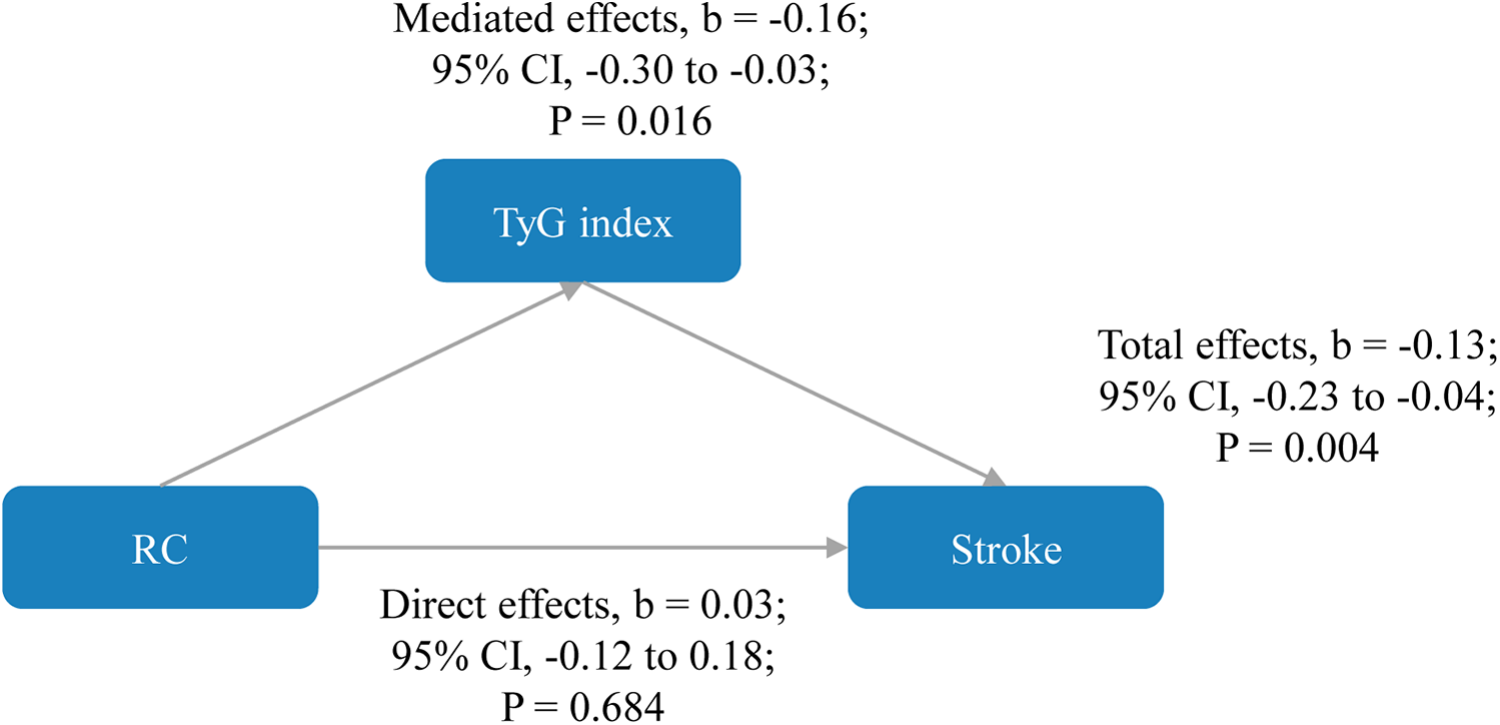
Mediated effects by triglyceride-glucose index on the associations of remnant cholesterol with stroke risk. The model was adjusted for age, gender, marital status, residence, education level, body mass index, smoking status, drinking status, diabetes, hypertension, heart disease, dyslipidemia, kidney disease, history of medication use for diabetes, history of medication use for hypertension, history of medication use for dyslipidemia, systole blood pressure, diastolic blood pressure, HbA1c, hsCRP, and eGFR. CI, confidence interval; RC, remnant cholesterol; TyG, triglyceride-glucose.

As shown in Figure 4, the direct effects of RC on stroke were not statistically significant (b, 0.03; 95% CI, −0.12 to 0.18; *P* = 0.684), while significant mediated effects by TyG index were observed on the associations between RC and stroke (b, −0.16; 95% CI, −0.30 to −0.03; *P* = 0.016). The results support that an increase in RC increases stroke risk through an increase in TyG index. Subgroup analyses is presented in Table 4, in participants aged ≥60 years, regardless of sex, marital status, rural residence, lack of formal education, body mass index between 18.5 and 23.9 kg/m^2^, smoking status (never smoked or current drinkers), and the presence or absence of hypertension, heart disease, diabetes, dyslipidemia, or kidney disease, similar results were observed. Similar results were also found when complete data analyses were conducted (Supplementary Figure S5). In addition, when employing TyG at Wave 3 to mitigate reverse causation, there were also significant mediated effects by TyG index (b, −0.10; 95% CI, −0.14 to −0.04; *P* < 0.001) as shown in Supplementary Figure S6. Finally, we analyzed whether RC mediated TyG index and stroke (Supplementary Figure S7). The results showed that no significant mediated effects by TC were observed on the associations between TyG index and stroke (b, 1.51; 95% CI, −4.81 to 8.37; *P* = 0.656).

**Table 4:** Associations of triglyceride-glucose index and remnant cholesterol with stroke risk stratified by different factors.

Subgroup	β (95% CI)^a^		
	Total effects	Direct effects	Mediated effects
Age, years			
<60	−0.16 (−0.23 to −0.10)	−0.11 (−0.22 to 0.10)	−0.05 (−0.21 to 0.02)
≥60	−0.13 (−0.24 to −0.06)	0.04 (−0.09 to 0.24)	−0.17 (−0.31 to −0.12)
Gender			
Male	−0.28 (−0.40 to −0.17)	−0.07 (−0.23 to 0.24)	−0.21 (−0.43 to −0.11)
Female	−0.08 (−0.15 to −0.04)	−0.03 (−0.12 to 0.11)	−0.05 (−0.16 to −0.01)
Marital status			
Married	−0.15 (−0.22 to −0.10)	−0.04 (−0.13 to 0.12)	−0.12 (−0.23 to −0.07)
Other	−0.20 (−0.39 to −0.04)	−0.11 (−0.37 to 0.31)	−0.09 (−0.38 to 0.03)
Residence			
Urban	−0.25 (−0.36 to −0.14)	−0.19 (−0.35 to 0.09)	−0.05 (−0.26 to 0.06)
Rural	−0.12 (−0.20 to −0.06)	0.03 (−0.09 to 0.20)	−0.15 (−0.27 to −0.10)
Education level			
No formal education	−0.06 (−0.21 to 0.15)	0.22 (−0.10 to 0.59)	−0.29 (−0.46 to −0.04)
Primary school	−0.02 (−0.12 to 0.13)	0.11 (−0.10 to 0.38)	−0.13 (−0.26 to 0.04)
Middle or high school	−0.12 (−0.23 to 0.02)	−0.04 (−0.26 to 0.25)	−0.08 (−0.25 to 0.15)
Body mass index, kg/m^2^			
18.5–23.9	−0.09 (−0.22 to 0.11)	0.21 (−0.09 to 0.54)	−0.30 (−0.45 to −0.08)
24.0–27.9	−0.00 (−0.11 to 0.16)	−0.01 (−0.27 to 0.30)	0.01 (−0.16 to 0.22)
≥28.0	−0.09 (−0.16 to 0.03)	−0.06 (−0.26 to 0.18)	−0.03 (−0.16 to 0.15)
Smoking status			
Never	−0.11 (−0.15 to −0.03)	0.03 (−0.07 to 0.13)	−0.14 (−0.25 to −0.04)
Former	−0.13 (−0.41 to 0.27)	0.16 (−0.32 to 0.59)	−0.28 (−0.69 to 0.12)
Current	−0.09 (−0.25 to 0.12)	0.03 (−0.29 to 0.33)	−0.12 (−0.40 to 0.15)
Drinking status			
Never	−0.17 (−0.23 to −0.06)	−0.03 (−0.16 to 0.10)	−0.13 (−0.28 to −0.00)
Former	0.27 (0.07 to 0.60)	0.38 (−0.03 to 0.74)	−0.11 (−0.44 to 0.23)
Current	−0.10 (−0.20 to 0.04)	0.13 (−0.09 to 0.35)	−0.23 (−0.44 to −0.04)
Hypertension			
No	−0.08 (−0.17 to 0.04)	0.06 (−0.12 to 0.26)	−0.14 (−0.34 to 0.02)
Yes	−0.15 (−0.22 to −0.07)	−0.05 (−0.15 to 0.13)	−0.10 (−0.22 to −0.06)
Without heart disease	−0.16 (−0.23 to −0.09)	0.01 (−0.09 to 0.18)	−0.18 (−0.29 to −0.13)
Without diabetes	−0.11 (−0.18 to −0.04)	0.04 (−0.06 to 0.22)	−0.15 (−0.27 to −0.11)
Without dyslipidemia	−0.15 (−0.22 to −0.07)	0.07 (−0.03 to 0.25)	−0.22 (−0.34 to −0.18)
Without kidney disease	−0.15 (−0.21 to −0.08)	−0.02 (−0.10 to 0.14)	−0.13 (−0.24 to −0.09)

CI, confidence interval.

^a^ Adjusted for age, gender, marital status, residence, education level, body mass index, smoking status, drinking status, diabetes, hypertension, heart disease, dyslipidemia, kidney disease, history of medication use for diabetes, history of medication use for hypertension, history of medication use for dyslipidemia, systole blood pressure, diastolic blood pressure, HbA1c, hsCRP, and eGFR. However, the stratification variable was not included.

## 4 Discussion

Among 10,569 Chinese adults aged 45 years or above followed up to 7.1 years, a higher baseline TyG index and RC were significantly associated with a higher risk of stroke. The highest risk of stroke onset was observed among those with a higher TyG index and RC. The associations persisted across subgroup analyses and sensitivity analyses. In addition, this study indicated that an increased TyG index completely mediated the association between RC and stroke.

The positive correlation between the TyG index and the risk of ischemic stroke has been extensively examined in previous research (16–19). In the Kailuan prospective cohort study (16), involving 96,541 Chinese participants, over a median follow-up period of 10.33 years, the TyG index exhibited an association with an elevated risk of cardiovascular diseases (CVD), encompassing myocardial infarction (MI), stroke, and ischemic stroke. Hong et al. (18) conducted a retrospective observational cohort study with 5,593,134 individuals aged over 40 years from 2009 to 2017, utilizing the National Health Information Database (NHID). Over an average follow-up period of 8.2 years, the study also revealed a significant association between a high TyG index and an increased risk of stroke. Similar results were also obtained in this study, suggesting that TyG index, an insulin resistance index, has a certain value in early prevention of stroke in high-risk populations.

In recent years, a growing body of research has shed light on the significant role that RC plays in the risk of stroke. RC, often referred to as non-HDL cholesterol, encompasses all cholesterol carried by atherogenic lipoproteins, including low-density lipoprotein (LDL) and very-low-density lipoprotein (VLDL) (11, 28). Several studies have underscored the importance of RC as a predictor of stroke risk, independent of traditional risk factors such as hypertension, smoking, and diabetes (3, 4, 29–32). The Copenhagen general population study found that step-wise higher remnant cholesterol concentrations were associated with step-wise higher ischemic stroke risk (4, 29). A Chinese cohort study has consistently demonstrated a positive correlation between higher RC levels and a higher incidence of ischemic stroke risk (3). Moreover, a prospective cohort study based on 1,956,452 patients with type 2 diabetes in Korea showed that elevated remnant cholesterol levels were associated with a 1.22-fold increased risk of ischemic stroke (30). Our study also confirms this conclusion, we found that a one SD increase in RC was associated with an elevated risk of stroke (HR, 1.09; 95% CI, 1.02–1.16). Therefore, the study on the association between RC, a non-traditional lipid index, and stroke has far-reaching significance for further guiding clinical practice.

Although, many studies have explored the relationship between RC and the TyG index and their association with stroke. However, to the best of our knowledge, this is the first study to investigate whether the TyG index mediates the association of RC with incident stroke and to what extent there is an interaction between RC and the TyG index in stroke occurrence. Our findings indicate that there is no significant synergistic interaction between these two factors in influencing stroke risk. Essentially, while both RC and the TyG index independently contribute to stroke risk, their combined effect does not surpass the sum of their individual impacts. Nevertheless, our results suggest that combining RC and the TyG index may offer greater predictive efficacy for stroke risk than either factor alone. The concept of joint effects implies that individuals with elevated levels in both RC and the TyG index might face a significantly higher stroke risk compared to those with high levels in only one of these measures. This insight has important implications for clinical practice and risk stratification. At last, Our results found that RC may not directly impact stroke risk, it may have an indirect influence through its relationship with the TyG index. Reverse analysis also showed that RC was not a mediator between TyG index and stroke. These findings underscore the complexity of risk factors for stroke and emphasize the importance of considering multiple variables when assessing and managing stroke risk in clinical practice. Further research may be needed to better understand the underlying mechanisms linking RC, the TyG index, and stroke risk.

There are possible mechanisms underlining the complex correlation among the TyG index or insulin resistance, RC, and stroke. Insulin resistance is a condition in which the body's cells do not respond effectively to insulin, a hormone responsible for regulating blood sugar (glucose) levels (33). When insulin resistance is present, the body may produce more insulin to compensate, leading to higher triglyceride levels and impaired glucose regulation (34). This dysregulation of glucose and lipids can contribute to inflammation and oxidative stress (35, 36), both of which are known to increase the risk of atherosclerosis and cardiovascular events, including stroke (37–41). Previous study has found that biomarker ApoB48 levels, reflecting intestinal RC and postprandial dyslipidemia, are positively correlated with insulin resistance (11, 42). Due to the short half-life of ApoB48-containing chylomicrons in normal blood tissue, which limits its clinical detection, RC can serve as an indirect indicator of chylomicron production levels. Previous studies have shown that increased production of chylomicrons is one of the main characteristics of insulin resistance and can lead to the development of atherosclerotic diseases by affecting triglyceride levels (43). Notably, this study found a strong positive correlation between RC and the TyG index, suggesting that RC can indirectly reflect the level of insulin resistance in patients with ischemic stroke.

Our findings have several clinical implications. First, they underscore the importance of considering the TyG index when assessing cardiovascular risk. Clinicians should be aware that individuals with elevated TyG index values may be at higher risk of stroke, particularly if they have RC. This information may help identify individuals who would benefit from more aggressive lipid-lowering and glucose-management strategies (44). Second, our results suggest that interventions aimed at improving insulin sensitivity and glycemic control could potentially mitigate the risk of stroke in individuals with high RC levels. Lifestyle modifications, such as dietary changes (45) and increased physical activity (46), should be encouraged in these patients. In some cases, pharmacological approaches to improve insulin sensitivity may also be considered (47, 48). Third, our study highlights the need for further research into the specific mechanisms by which the TyG index mediates the association between RC and stroke. Understanding these mechanisms at the molecular and cellular levels could lead to the development of targeted therapies that address both lipid and glucose dysregulation to reduce stroke risk effectively.

The strengths of this study included the prospective design and the mediation analyses. However, several limitations of this study warrant acknowledgment. First, given its observational nature, we cannot establish a causal association between RC, the TyG index, and stroke risk. Nonetheless, we conducted sensitivity analyses to mitigate potential reverse causal relationships and consistently obtained confirmatory results. Second, despite adjusting for potential stroke risk factors, the possibility of residual or unmeasured confounding cannot be entirely ruled out due to the study's observational design, which includes factors such as diet (45), physical activity (46), and family history of stroke (49). Third, the diagnosis of stroke events relied on self-reporting, as medical records were unavailable within the CHARLS dataset. However, it is worth noting that other extensive studies, such as the English Longitudinal Study of Aging, have shown good agreement between self-reported incident cardiovascular disease and medical records (50). Last, the CHARLS predominantly comprises Chinese participants aged 45 years and older; thus, the observed associations in this study may not be fully generalizable to broader populations or different disease contexts. Further investigations are warranted to validate these findings in diverse populations and health conditions.

## 5 Conclusions

In this prospective cohort study involving Chinese adults, we have identified a significant mediating role of insulin resistance, as represented by the TyG index, in the relationship between RC and stroke. These findings advocate for a comprehensive evaluation that incorporates both the TyG index and RC in order to more effectively stratify the risk of stroke.

## Data Availability

Publicly available datasets were analyzed in this study. This data can be found here: https://charls.pku.edu.cn.
