# Supplementary material for: Triglyceride-glucose index mediates the association between residual cholesterol and stroke among middle-aged and older adults in China: a prospective, nationwide, population-based study
Source: Front Cardiovasc Med. 2024 Dec 5;11:1429993. doi: 10.3389/fcvm.2024.1429993 (PMC11660090; doi:10.3389/fcvm.2024.1429993)
Supplement: Supplementary file 1 [file Datasheet1.pdf]

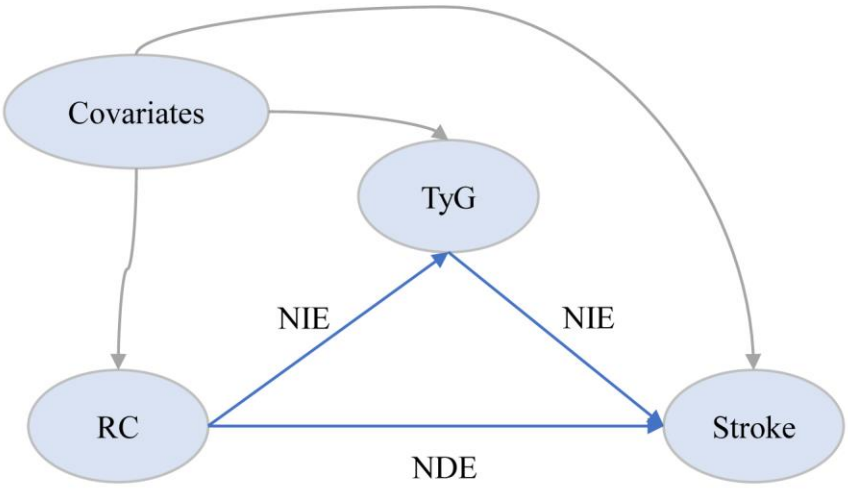

Figure S1. Mediating pathway of the association of remnant cholesterol with stroke. Direct acyclic graph of a structural model of mediation of the association between remnant cholesterol and stroke by triglyceride-glucose index. **Abbreviations:** RC, remnant cholesterol; TyG, triglyceride-glucose; NDE, natural direct effects; NIE, natural indirect effects.

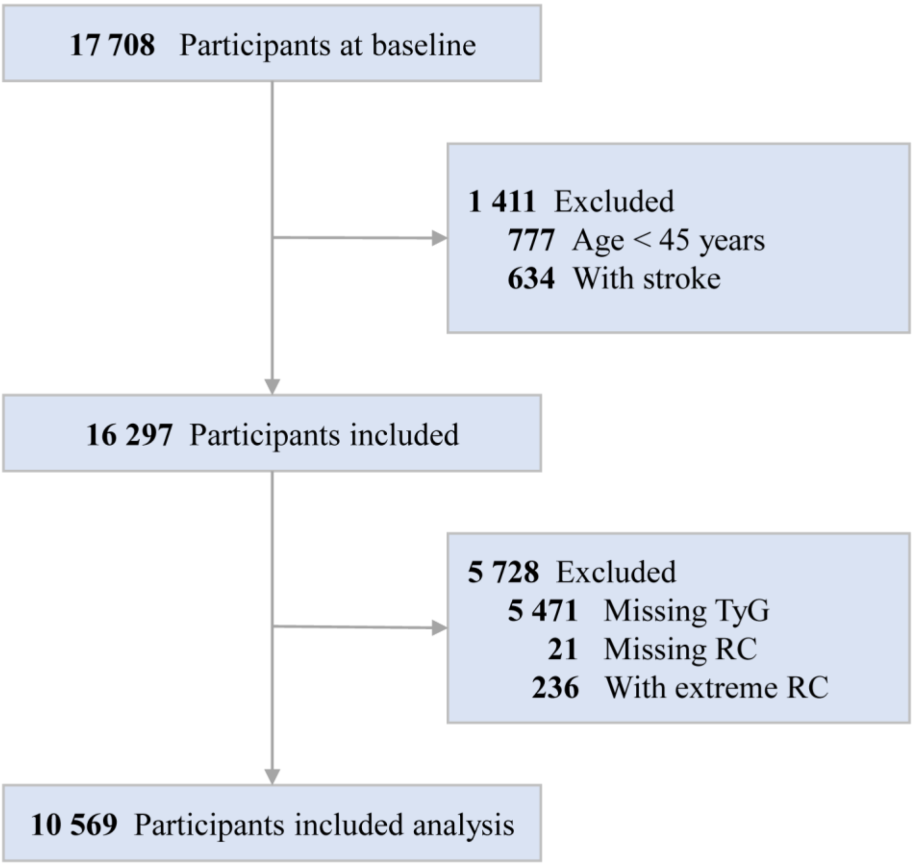

Figure S2. Flowchart of the study population.

Abbreviations: RC, remnant cholesterol; TyG, triglyceride-glucose.

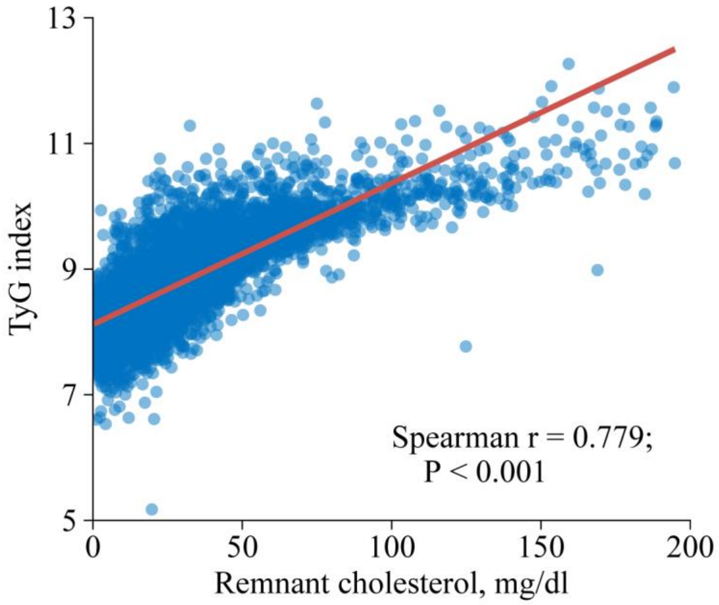

Figure S3. Spearman correlation analysis of remnant cholesterol and triglyceride-glucose index in subpopulations of 8 515 participants with complete data.

Abbreviations: TyG, triglyceride-glucose.

TyG index	RC	No. of event	HR (95% CI)	P value
Q1	Q1	60	Reference	
Q1	Q2	26	1.07 (0.67–1.70)	0.777
Q1	Q3	9	0.89 (0.44–1.79)	0.737
Q1	Q4	2	1.83 (0.44–7.56)	0.406
Q2	Q1	32	1.09 (0.71–1.68)	0.686
Q2	Q2	68	1.55 (1.10–2.20)	0.013
Q2	Q3	31	1.37 (0.89–2.12)	0.157
Q2	Q4	8	1.57 (0.75–3.29)	0.232
Q3	Q1	15	1.88 (1.06–3.33)	0.031
Q3	Q2	53	1.60 (1.10–2.32)	0.015
Q3	Q3	77	1.51 (1.07–2.13)	0.018
Q3	Q4	38	1.67 (1.11–2.52)	0.014
Q4	Q1	2	0.91 (0.22–3.86)	0.902
Q4	Q2	2	0.32 (0.08–1.35)	0.121
Q4	Q3	35	1.27 (0.82–1.96)	0.288
Q4	Q4	145	1.58 (1.15–2.16)	0.005

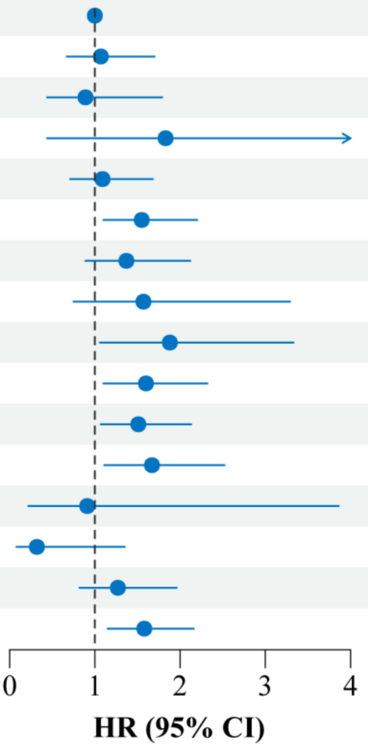

Synergy index, 1.05; 95% CI, 0.16 to 6.88

HR for multiplicative, 1.07; 95% CI, 0.67 to 1.70

Figure S4. Interaction and joint effects for triglyceride-glucose index and remnant cholesterol on stroke risk in subpopulations of 8 515 participants with complete data. Graphs show the interaction and joint effects of the TyG index and remnant cholesterol with stroke risk. The model was adjusted for age, gender, marital status, residence, education level, body mass index, smoking status, drinking status, diabetes, hypertension, heart disease, dyslipidemia, kidney disease, history of medication use for diabetes, history of medication use for hypertension, history of medication use for dyslipidemia, systole blood pressure, diastolic blood pressure, HbA1c, hsCRP, and eGFR. Additive interaction was evaluated using synergy index (SI) between the TyG index and remnant cholesterol, and the additive interaction was statistically significant when its CI did not include 1. Multiplicative interaction was evaluated using HR for the product term between the TyG index and remnant cholesterol, and the multiplicative interaction was statistically significant when its CI did not include 1.

Abbreviations: CI, confidence interval; HR, hazard ratio; Q, quartile, RC, remnant cholesterol; TyG, triglyceride-glucose.

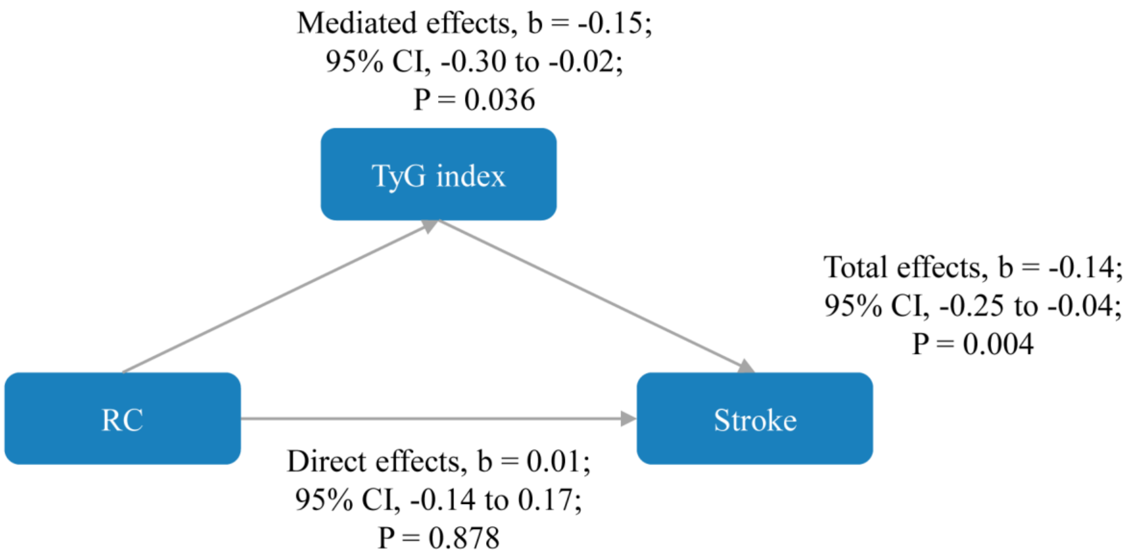

Figure S5. Mediated effects by triglyceride-glucose index on the associations of remnant cholesterol with stroke risk in subpopulations of 8 515 participants with complete data. The model was adjusted for age, gender, marital status, residence, education level, body mass index, smoking status, drinking status, diabetes, hypertension, heart disease, dyslipidemia, kidney disease, history of medication use for diabetes, history of medication use for hypertension, history of medication use for dyslipidemia, systole blood pressure, diastolic blood pressure, HbA1c, hsCRP, and eGFR. **Abbreviations:** CI, confidence interval; RC, remnant cholesterol; TyG, triglyceride-glucose.

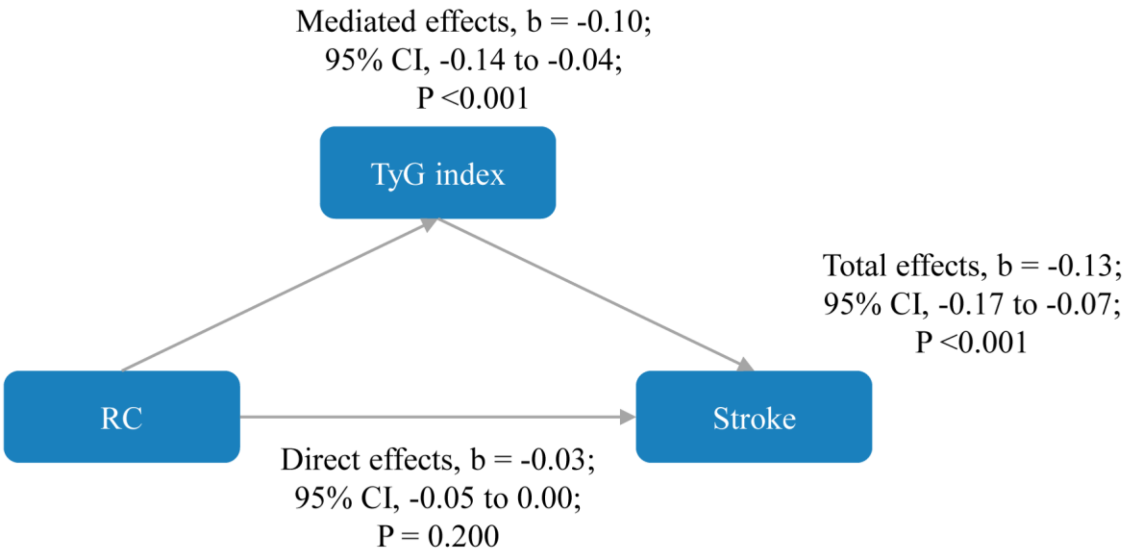

Figure S6. Mediated effects by triglyceride-glucose index at Wave 3 on the associations of remnant cholesterol with stroke risk

The model was adjusted for age, gender, marital status, residence, education level, body mass index, smoking status, drinking status, diabetes, hypertension, heart disease, dyslipidemia, kidney disease, history of medication use for diabetes, history of medication use for hypertension, history of medication use for dyslipidemia, systole blood pressure, diastolic blood pressure, HbA1c, hsCRP, and eGFR. **Abbreviations:** CI, confidence interval; RC, remnant cholesterol; TyG, triglyceride-glucose.

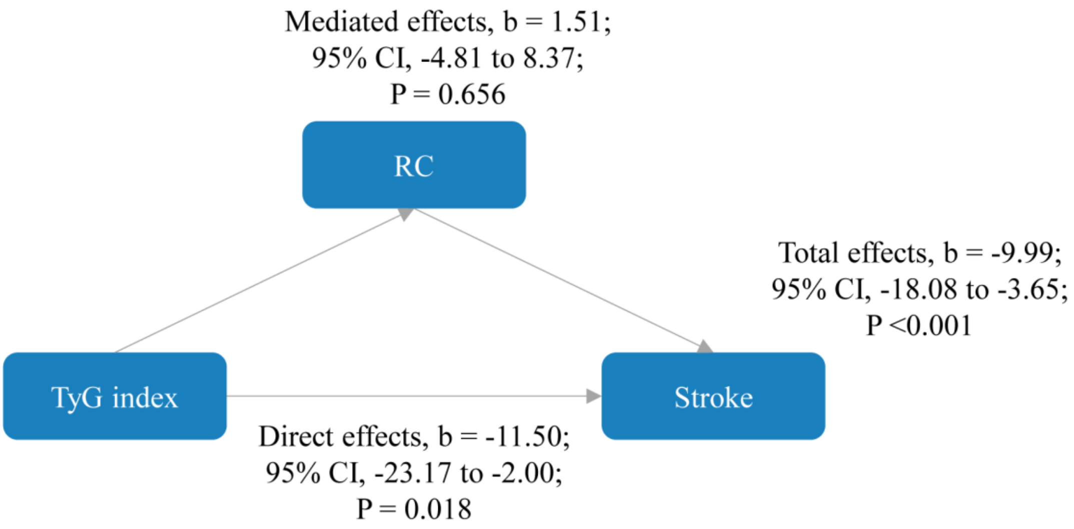

Figure S7. Mediated effects by remnant cholesterol on the associations of triglyceride-glucose index with stroke risk.

The model was adjusted for age, gender, marital status, residence, education level, body mass index, smoking status, drinking status, diabetes, hypertension, heart disease, dyslipidemia, kidney disease, history of medication use for diabetes, history of medication use for hypertension, history of medication use for dyslipidemia, systole blood pressure, diastolic blood pressure, HbA1c, hsCRP, and eGFR. **Abbreviations:** CI, confidence interval; RC, remnant cholesterol; TyG, triglyceride-glucose.

Table S1. Baseline characteristics between participants included and not included

Characteristic	Exclude (n=7139)	Include (n=10569)	P value [a]
Age, years			<0.001
<60	4241 (59.4%)	6002 (56.8%)	
≥60	2723 (38.1%)	4567 (43.2%)	
Missing	175 (2.5%)	0 (0.0%)	
Gender			0.011
Male	3500 (49.0%)	4978 (47.1%)	
Female	3637 (50.9%)	5591 (52.9%)	
Missing	2 (0.0%)	0 (0.0%)	
Marital status			<0.001
Married	5434 (76.1%)	8736 (82.7%)	
Other	1693 (23.7%)	1833 (17.3%)	
Missing	12 (0.2%)	0 (0.0%)	
Residence			<0.001
Urban	3310 (46.4%)	3861 (36.5%)	
Rural	3829 (53.6%)	6708 (63.5%)	
Education level			<0.001
No formal education	1775 (24.9%)	3076 (29.1%)	
Primary school	2685 (37.6%)	4235 (40.1%)	
Middle or high school	2229 (31.2%)	2874 (27.2%)	
College or above	434 (6.1%)	384 (3.6%)	
Missing	16 (0.2%)	0 (0.0%)	
Smoking status			<0.001
Never	4232 (59.3%)	6387 (60.4%)	
Former	534 (7.5%)	883 (8.4%)	
Current	1782 (25.0%)	3089 (29.2%)	
Missing	591 (8.3%)	210 (2.0%)	
Body mass index, kg/m^2			0.160
<18.5	339 (4.7%)	610 (5.8%)	
18.5-23.9	2517 (35.3%)	4677 (44.3%)	
24.0-27.9	1314 (18.4%)	2628 (24.9%)	
≥28.0	510 (7.1%)	1036 (9.8%)	
Missing	2459 (34.4%)	1618 (15.3%)	
Drinking status			0.573
Never	4146 (58.1%)	6187 (58.5%)	
Former	574 (8.0%)	869 (8.2%)	
Current	2265 (31.7%)	3502 (33.1%)	
Missing	154 (2.2%)	11 (0.1%)	
Hypertension			0.039

Characteristic	Exclude (n=7139)	Include (n=10569)	P value [a]
No	5208 (73.0%)	5253 (49.7%)	
Yes	1747 (24.5%)	4193 (39.7%)	
Missing	184 (2.6%)	1123 (10.6%)	
Diabetes			0.893
No	6514 (91.2%)	9832 (93.0%)	
Yes	421 (5.9%)	641 (6.1%)	
Missing	204 (2.9%)	96 (0.9%)	
Heart disease			0.856
No	6089 (85.3%)	9248 (87.5%)	
Yes	850 (11.9%)	1280 (12.1%)	
Missing	200 (2.8%)	41 (0.4%)	
Dyslipidemia			0.499
No	6188 (86.7%)	9329 (88.3%)	
Yes	662 (9.3%)	1034 (9.8%)	
Missing	289 (4.0%)	206 (1.9%)	
Kidney disease			0.080
No	6572 (92.1%)	9901 (93.7%)	
Yes	363 (5.1%)	616 (5.8%)	
Missing	204 (2.9%)	52 (0.5%)	
History of medication use for hypertension			0.331
No	5616 (78.7%)	8430 (79.8%)	
Yes	1334 (18.7%)	2080 (19.7%)	
Missing	189 (2.6%)	59 (0.6%)	
History of medication use for diabetes			0.294
No	6635 (92.9%)	10057 (95.2%)	
Yes	297 (4.2%)	415 (3.9%)	
Missing	207 (2.9%)	97 (0.9%)	
History of medication use for dyslipidemia			0.843
No	6498 (91.0%)	9838 (93.1%)	
Yes	347 (4.9%)	518 (4.9%)	
Missing	294 (4.1%)	213 (2.0%)	
Systole blood pressure, mmHg			0.863
Mean±SD	129.54±21.74	129.61±21.46	
Missing	2378 (33.3%)	1590 (15.0%)	
Diastolic blood pressure, mmHg			0.105
Mean±SD	75.72±12.32	75.36±12.22	
Missing	2378 (33.3%)	1591 (15.0%)	
FBG, mg/dl			0.869
Mean±SD	110.10±41.25	110.32±36.92	
Missing	6072 (85.1%)	0	

Characteristic	Exclude (n=7139)	Include (n=10569)	P value [a]
TC, mg/dl			0.036
Mean±SD	190.13±47.50	193.26±37.89	
Missing	6053 (84.8%)	0	
TG, mg/dl			<0.001
Median (IQR)	97.35 (66.38, 158.41)	107.08 (76.11, 156.65)	
Missing	6052 (84.8%)	0	
HDL-C, mg/dl			0.948
Mean±SD	50.88±18.10	50.84±15.01	
Missing	6045 (84.7%)	0	
LDL-C, mg/dl			<0.001
Mean±SD	110.79±35.85	116.52±34.77	
Missing	6066 (85.0%)	0	
hs-CRP, mg/l			0.011
Median (IQR)	0.99 (0.48, 2.12)	1.04 (0.56, 2.21)	
Missing	6044 (84.7%)	0	
HbA1c, %			0.003
Mean±SD	5.20±0.82	5.27±0.82	
Missing	5910 (82.8%)	92	
TyG index [b]			0.250
Mean±SD	8.67±0.93	8.70±0.65	
Missing	6072 (85.1%)	0	
RC, mg/dl [c]			<0.001
Median (IQR)	16.62 (5.80, 30.25)	20.10 (11.98, 32.09)	
Missing	6079 (85.2%)	0	

Data were presented as n(%), unless otherwise specified.

Abbreviations: FBG: fast blood glucose; HbA1c: glycated hemoglobin; HDL-C: high-density lipoprotein cholesterol; hsCRP, high-sensitivity C-reactive protein; IQR: interquartile range; LDL-C: low-density lipoprotein cholesterol; RC, remnant cholesterol; SD: standard deviation; TC: total cholesterol; TyG: triglyceride-glucose.

[a] P value was based on the χ^2 test, Mann-Whitney U test, or analysis of variance test where appropriate

[b] TyG index was calculated by the formula ln[Triglyceride (mg/dl) × Fasting blood glucose (mg/dl)/2].

[c] RC was calculated by the formula TC (mg/dl) – LDL-C (mg/dl) – HDL-C (mg/dl).

Table S2. Associations of triglyceride-glucose index and remnant cholesterol with stroke risk by competing risk analysis

Variables	No. of event / total	Model 1 [a]		Model 2 [b]		Model 3 [c]	
		HR (95% CI)	P value	HR (95% CI)	P value	HR (95% CI)	P value
TyG index							
Quartile							
Q1 [5.18, 8.25]	120 / 2643	Reference		Reference		Reference	
Q2 (8.25, 8.62]	157 / 2642	1.31 (1.04–1.66)	0.024	1.26 (1.00–1.60)	0.053	1.22 (0.96–1.55)	0.100
Q3 (8.62, 9.07]	205 / 2642	1.73 (1.38–2.17)	<0.001	1.56 (1.24–1.96)	<0.001	1.41 (1.12–1.78)	0.003
Q4 (9.07, 12.3]	225 / 2642	1.93 (1.55–2.41)	<0.001	1.66 (1.32–2.09)	<0.001	1.39 (1.10–1.77)	0.006
Per-1-SD increase	707 / 10569	1.27 (1.19–1.36)	<0.001	1.21 (1.13–1.29)	<0.001	1.13 (1.05–1.22)	0.001
Remnant cholesterol							
Quartile							
Q1 [0.39, 12.00]	135 / 2647	Reference		Reference		Reference	
Q2 (12.00, 20.10]	174 / 2643	1.34 (1.08–1.68)	0.009	1.29 (1.03–1.61)	0.027	1.25 (1.00–1.56)	0.052
Q3 (20.10, 32.10]	169 / 2642	1.31 (1.05–1.64)	0.018	1.21 (0.97–1.52)	0.096	1.15 (0.92–1.44)	0.232
Q4 (32.10, 195.00]	229 / 2637	1.82 (1.48–2.25)	<0.001	1.57 (1.27–1.95)	<0.001	1.43 (1.15–1.77)	0.001
Per-1-SD increase	707 / 10569	1.18 (1.12–1.24)	<0.001	1.13 (1.07–1.20)	<0.001	1.08 (1.02–1.15)	0.007

Abbreviations: CI, confidence interval; HR, hazard ratio; SD, standard deviation; TyG, triglyceride-glucose; Q, quartile.

[a] Adjusted for age and gender.

[b] Adjusted for age, gender, marital status, residence, education level, body mass index, smoking status, and drinking status.

[c] Adjusted as model 2 plus diabetes, hypertension, heart disease, dyslipidemia, kidney disease, history of medication use for diabetes, history of medication use for hypertension, history of medication use for dyslipidemia, systole blood pressure, diastolic blood pressure, HbA1c, hsCRP, and eGFR.

Table S3. Associations of triglyceride-glucose index and remnant cholesterol with stroke risk in subpopulations of 8 515 participants with complete data

Variables	No. of event / total	Model 1 [a]		Model 2 [b]		Model 3 [c]	
		HR (95% CI)	P value	HR (95% CI)	P value	HR (95% CI)	P value
TyG index							
Quartile							
Q1 [5.18, 8.25]	97 / 2180	Reference		Reference		Reference	
Q2 (8.25, 8.62]	139 / 2128	1.47 (1.13–1.91)	0.004	1.41 (1.09–1.83)	0.009	1.36 (1.05–1.76)	0.022
Q3 (8.62, 9.07]	183 / 2124	1.96 (1.53–2.52)	<0.001	1.75 (1.36–2.25)	<0.001	1.57 (1.22–2.02)	<0.001
Q4 (9.07, 12.3]	184 / 2083	2.06 (1.61–2.64)	<0.001	1.75 (1.36–2.26)	<0.001	1.44 (1.10–1.88)	0.007
Per-1-SD increase	603 / 8515	1.30 (1.21–1.40)	<0.001	1.23 (1.14–1.33)	<0.001	1.15 (1.06–1.26)	0.001
Remnant cholesterol							
Quartile							
Q1 [0.39, 12.00]	109 / 2123	Reference		Reference		Reference	
Q2 (12.00, 20.10]	149 / 2161	1.38 (1.08–1.77)	0.011	1.32 (1.03–1.69)	0.030	1.26 (0.98–1.62)	0.066
Q3 (20.10, 32.10]	152 / 2118	1.45 (1.13–1.85)	0.003	1.32 (1.03–1.70)	0.027	1.24 (0.97–1.60)	0.085
Q4 (32.10, 195.00]	193 / 2113	1.90 (1.51–2.41)	<0.001	1.63 (1.28–2.07)	<0.001	1.45 (1.14–1.85)	0.003
Per-1-SD increase	603 / 8515	1.20 (1.13–1.28)	<0.001	1.15 (1.08–1.23)	<0.001	1.10 (1.03–1.18)	0.005

Abbreviations: CI, confidence interval; HR, hazard ratio; SD, standard deviation; TyG, triglyceride-glucose; Q, quartile.

[a] Adjusted for age and gender.

[b] Adjusted for age, gender, marital status, residence, education level, body mass index, smoking status, and drinking status.

[c] Adjusted as model 2 plus diabetes, hypertension, heart disease, dyslipidemia, kidney disease, history of medication use for diabetes, history of medication use for hypertension, history of medication use for dyslipidemia, systole blood pressure, diastolic blood pressure, HbA1c, hsCRP, and eGFR.
